# *GJB6* missense variant in a Labrador Retriever with paw pad hyperkeratosis

**DOI:** 10.1002/age.70075

**Published:** 2026-01-27

**Authors:** Stefan J. Rietmann, Joseph Malatos, Vidhya Jagannathan, Tosso Leeb

**Affiliations:** ^1^ Institute of Genetics, Vetsuisse Faculty University of Bern Bern Switzerland; ^2^ IDEXX Laboratories Westbrook Maine USA

**Keywords:** *Canis lupus familiaris*, dermatology, dog, footpad, precision medicine, skin, whole genome sequencing

## Abstract

Palmoplantar keratoderma in humans is a condition defined by an abnormally thickened cornified skin layer on the hands and feet. In animals, the corresponding disease is commonly termed paw pad hyperkeratosis. It can be acquired due to repeated trauma, infections, cancer, or inflammatory dermatoses, or inherited due to pathogenic variants in genes involved in skin development. More than 60 different genes involved in the development of palmoplantar keratoderma have been described. Here, we investigated a female Labrador Retriever showing hyperkeratosis on all four paw pads and most digital pads. Histologically, the stratum corneum was expanded by predominantly orthokeratotic hyperkeratosis with occasional mild parakeratotic areas. DNA of the affected dog was isolated from EDTA‐blood and whole genome sequencing was performed. Comparison of the whole genome sequencing data to 1664 unaffected control dogs revealed a private de novo heterozygous missense variant in the *GJB6* gene which was not present in the parents. *GJB6* encodes connexin 30, a subunit of the desmosome. In humans, pathogenic variants in this gene cause isolated deafness or Clouston syndrome, an autosomal dominant condition that is characterized by alopecia, nail dystrophy, and palmoplantar hyperkeratosis. The paw pad hyperkeratosis phenotype in the investigated dog shows similarities to Clouston syndrome and strongly suggests that the *GJB6* missense variant is responsible for its condition. However, our investigation also highlights differences between human and dog that could provide deeper insights into the function of *GJB6*.

## INTRODUCTION

The skin covers the entire surface of the body and acts as a barrier between the organism and its environment, making it subject to a broad range of external stressors (Mauldin & Elias, 2021). Paw pads in animals are particularly exposed to physical stress, and many species have evolved specific adaptations in this area, such as a thickened cornified epidermal layer (Boyle et al., 2019). Hyperplasia of the palmoplantar epidermis results in a condition referred to as palmoplantar keratoderma (PPK) in humans (Sakiyama & Kubo, 2016). In quadrupedal animals, the homologous condition is commonly termed paw pad hyperkeratosis.

While most cases of human PPK are acquired due to repeated trauma, infections, cancer, or inflammatory dermatoses, they can also occur as an inherited condition (Bodemer et al., 2021). Pathogenic variants causing PPK have been reported in genes encoding keratins, subunits of the desmosomes or gap junctions, water channels, and epidermal growth factor receptor signaling molecules (Has & Technau‐Hafsi, 2016). So far, over 60 candidate genes for PPK have been identified in humans (Table S1). PPK may follow autosomal dominant, autosomal recessive, or X‐chromosomal patterns of inheritance (Guerra et al., 2018a, 2018b; https://panelapp.genomicsengland.co.uk/panels/556/, accessed 08. Aug. 2025).

Hereditary PPKs are further categorized into isolated forms, which are confined to the skin of the palms and soles, and syndromic forms, which are associated with additional lesions affecting other organs (Has & Technau‐Hafsi, 2016).

In animals, so far, only three genetically distinct forms of paw pad hyperkeratosis have been characterized at the molecular level. An autosomal recessive form caused by a *FAM83G*:p.R52P missense variant was reported in Kromfohrländer dogs and Irish Terriers (Drögemüller et al., 2014; OMIA 001327–9615). Another autosomal recessive form of paw pad hyperkeratosis combined with severe atopic dermatitis was identified in a single Rottweiler with a homozygous frameshift variant in *DSG1* (Backel et al., 2020; OMIA 002266–9615). Finally, an autosomal dominant epidermolytic ichthyosis combined with paw pad hyperkeratosis occurred in a Chihuahua carrying a heterozygous *KRT10*:R146H missense variant (Kiener et al., 2023; OMIA 001415–9615).

In this study, we investigated a 2‐year‐old female Labrador Retriever with thickened, spike‐like epidermis on all four paw pads. The aim of the study was to characterize the clinical and histopathological phenotype and to determine a potential causal genetic variant.

## METHODS

### Animal selection

Samples from 91 Labrador Retrievers from the Vetsuisse Biobank were used in this study. They included the dog affected with paw pad hyperkeratosis, its parents, and 88 additional Labrador Retrievers without known close relationships to the affected dog. None of the other dogs showed signs of paw pad hyperkeratosis, although 13 of them were affected by atopic dermatitis (Table S2). Additionally, whole genome sequencing (WGS) data from 1664 other dogs of various breeds were used during variant filtering.

### Clinical and histopathological examinations

The paw pad hyperkeratosis‐affected Labrador Retriever was repeatedly examined at the same private veterinary clinic. Two incisional punch biopsies of the affected areas were obtained, routinely processed, stained with hematoxylin and eosin, and examined by a board‐certified veterinary anatomic pathologist (J.M.).

### DNA extraction & whole genome sequencing

Genomic DNA was extracted from EDTA‐whole blood samples. The extraction was performed with a Maxwell™ RSC instrument using the Maxwell RSC Whole Blood DNA Kit (Promega, Dübendorf, Switzerland). For whole genome sequencing of the affected dog, a PCR‐free library with an insert size of approximately 365 bp was produced and sequencing was accomplished with 2 × 150‐bp reads on an Illumina NovaSeq™ 6000 sequencing system at 32.9× coverage (Illumina, Zürich, Switzerland). The resulting raw data were mapped to the UU_Cfam_GSD_1.0 reference genome assembly using a previously published workflow (Jagannathan et al., 2019).

### Variant calling and filtering

For the identification of single nucleotide variants and small indel variants (<20 bp), we used the GATK Haplotype Caller software (McKenna et al., 2010) in gVCF mode as previously described (Jagannathan et al., 2019). Functional effects of the variants were annotated with SnpEff software in combination with NCBI annotation release 106 (Cingolani et al., 2012). For identification of variants private to the case, the WGS data of the paw pad hyperkeratosis affected Labrador were filtered against a control cohort of 1664 dogs of various breeds. All variants present in at least one other dog were excluded. Variants with a predicted effect of “high” or “moderate” were considered protein changing and checked for known candidate genes associated with palmoplantar keratoderma (Table S1). Accession numbers of the WGS data are given in Table S3.

### PCR amplification and sanger sequencing

A primer pair flanking the identified XM_038573516.1:c.223C>T missense variant was designed for PCR amplification of the region of interest. Primer sequences were 5′‐TCCTTGTAGTGGCTGCTCAA‐3′ and 5′‐CCACATTTCAACACCCAGGG‐3′. A 406‐bp PCR product was produced from genomic DNA samples using AmpliTaqGold™ 360MasterMix with 30 amplification cycles (Thermo Fisher Scientific, Waltham, MA, USA). PCR products were purified using exonuclease I and alkaline phosphatase.

The reverse primer was used for subsequent Sanger sequencing of the purified PCR products using the Applied Biosystems™ BigDye™ v3.1 cycle sequencing kit on an Applied Biosystems™ 3730 DNA Analyzer (Thermo Fisher Scientific). The resulting data were visualized with the Sequencher™ 5.1 software (Gene Codes Corporation, Ann Arbor, MI USA).

### In silico protein analyses

The potential impact of the variant XP_038429444.1:p.Arg75Trp was assessed using the prediction tools PredictSNP, SNPs&Go and MutPred2 (Bendl et al., 2014; Calabrese et al., 2009; Pejaver et al., 2020).

### Parentage verification

Sixteen microsatellite markers from the ISAG 2006 recommended set were successfully genotyped in the affected dog and its reported parents by an external service provider (Laboklin, Bad Kissingen, Germany). The resulting genotypes are given in Table S4.

## RESULTS

### Clinical examination

The investigated female dog showed no abnormalities at birth and developed normally. The patient was spayed at age around 1 year with no reported adverse events during the surgical procedure and subsequent recovery. At age approximately 16 months, the owner noticed a decreased or inappropriate reaction to commands or verbal clues and expressed suspicion that the patient may have deafness or impaired hearing function. No further investigation was pursued and to this date no clear diagnosis was made.

**FIGURE 1:**
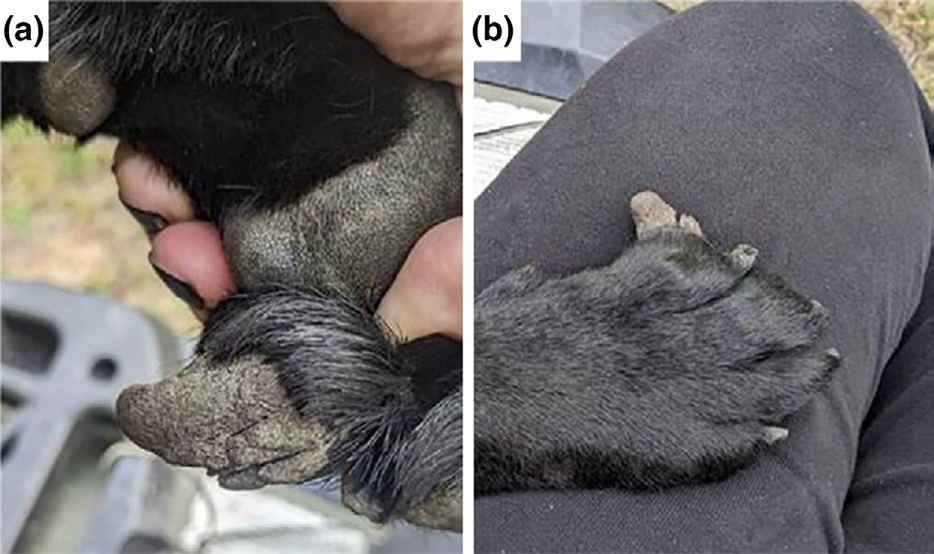
Clinical phenotype of the affected dog. (a) Excessive thick projection on one of the outer digit pads before surgical intervention. Splits in the paw pad skin can be seen in the hyperkeratotic projection. (b) Dorsal view of the paw.

At approximately 18 months old, the patient was presented to a veterinarian due to an abnormal growth on one of her outer toes (Figure 1). Upon further examination by a veterinarian, excessive skin growth was observed present on all four paw pads and most of the digital pads to some degree. The medial aspect of the right front second digit was most severely affected. The areas appeared non painful and superficial splits were present in some of them. On veterinary recommendation the owner started regularly soaking the paw pads in a 50/50 propylene glycol–water solution, which did not lead to any significant improvement. A surgical intervention was performed to take biopsies of the affected areas and remove some of the abnormal growth.

### Histopathological examination

**FIGURE 2:**
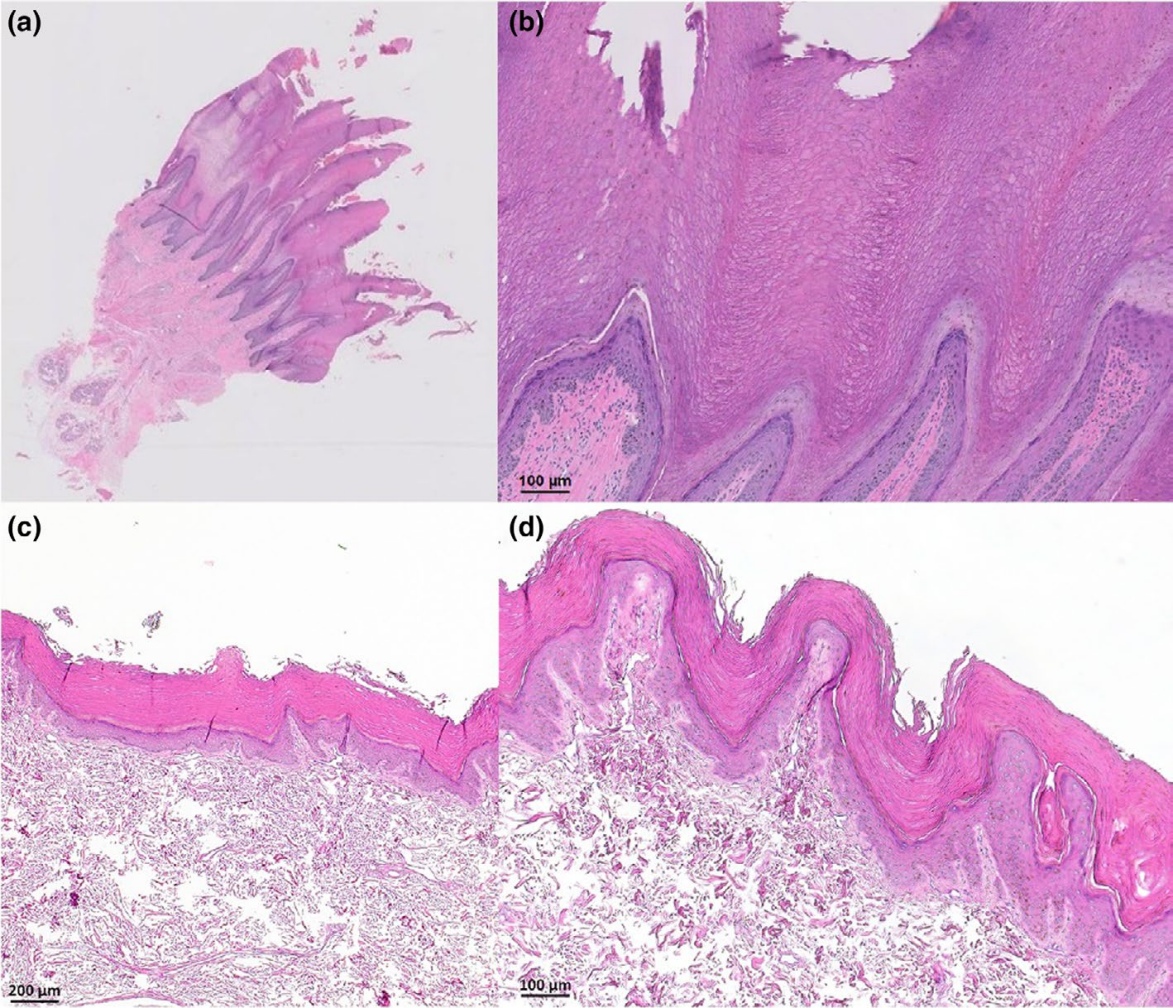
Histopathological findings in a paw pad biopsy from an affected dog. (a, b) Affected dog. (c, d) Unaffected control dog. (a) The epidermis is mildly hyperplastic and covered by a thickened layer of compact keratin forming villous‐like projections. Hematoxylin and eosin, 0.5×. (b) The stratum corneum is expanded by predominately compact orthokeratotic hyperkeratosis. Hematoxylin and eosin, 10×.

Histological examination revealed a stratum corneum expanded by diffuse moderate to severe orthokeratotic and occasional mild parakeratotic hyperkeratosis arranged in spires or villous‐like projections (Figure 2). The epidermis was mildly hyperplastic, and there were small numbers of perivascular lymphocytes, plasma cells, and mast cells in the superficial dermis.

### Genetic analysis

To identify a candidate causal genetic variant, we performed whole genome sequencing of the affected dog. Filtering for private variants against 1664 control genomes resulted in the identification of 10 heterozygous and 2 homozygous private protein changing variants (Table 1). All identified private variants and their predicted effects can be found in Table S5.

**TABLE 1:** Variants identified in the affected dog and filtering against 1664 control genomes.

Filtering step	Heterozygous variants	Homozygous variants
All variants	3 347 102	2 991 782
Private variants	1074	254
Private protein changing variants	10	2
Private protein changing variants in a candidate gene^a^	1	0

^a^ Candidate genes are listed in Table S1.

**FIGURE 3:**
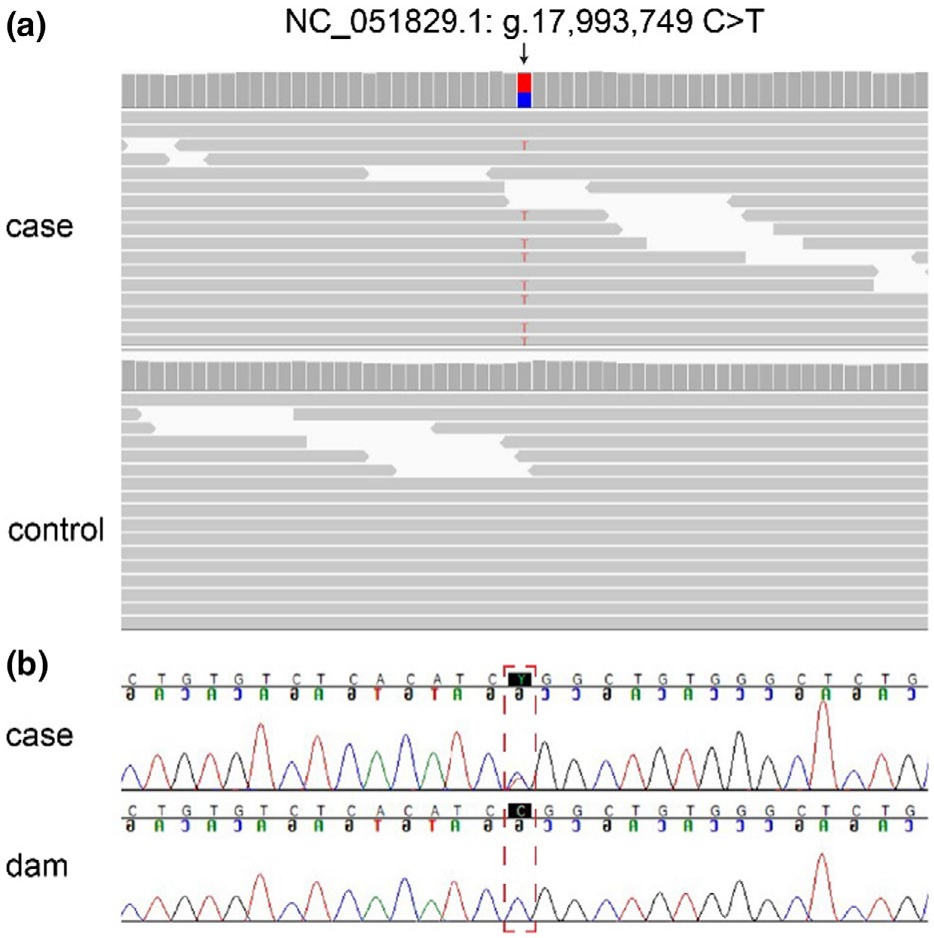
Details of the *GJB6*:XM_038573516.1:C.223C>T variant. (a) Short read‐alignments of the affected dog and a control dog harboring the *GJB6* missense variant depicted in the integrative genomics viewer (IGV; Thorvaldsdóttir et al., 2012). (b) Sanger sequencing results of the affected dog and its dam. The position of the *GJB6* missense variant is highlighted with a red dotted square. The case is heterozygous at the position while the dam is homozygous for the wildtype genotype.

Only one of the remaining private variants was located in a functional candidate gene for paw pad hyperkeratosis (Table S1). This was a heterozygous missense variant in the *GJB6* gene NC_051829.1:g.17993749 C>T or XM_038573516.1:c.223C>T (Figure 3).

We genotyped the parents and 88 other Labrador Retrievers for the identified *GJB6* variant. None of them carried the mutant allele. The parentage of the alleged parents was experimentally corroborated by genotyping 16 microsatellites that all showed concordant genotypes in the trio (Table S4). These genotyping results confirmed that the *GJB6* variant emerged from a de novo mutation event.

**FIGURE 4:**
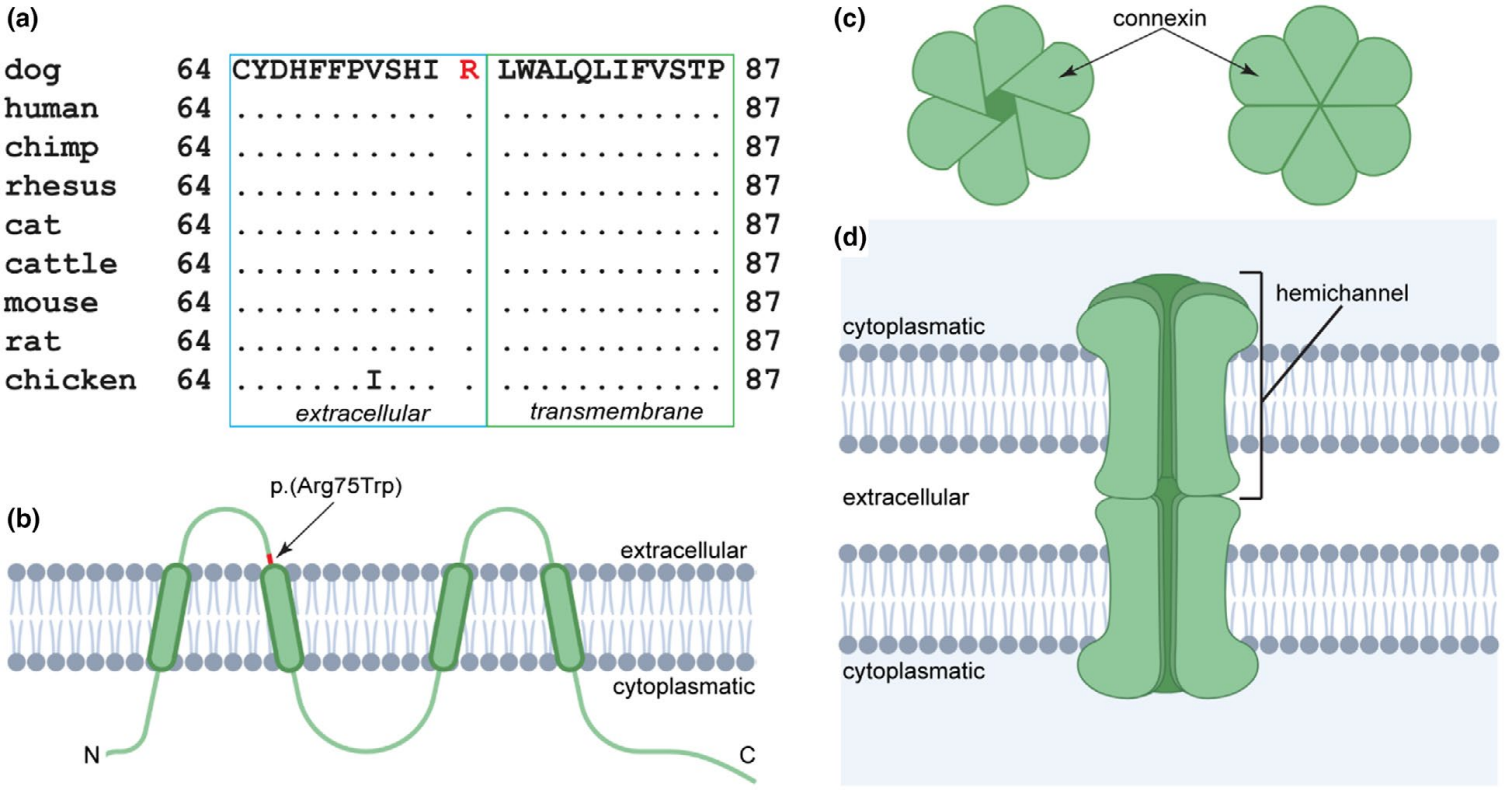
GJB6 protein and structure of a gap junction. (a) The exchanged amino acid (highlighted in red) is evolutionarily conserved among species. (b) GJB6 is an integral membrane protein with four transmembrane domains and cytoplasmic N‐ and C‐termini. The identified amino acid exchange affects the last position of the first extracellular loop (Kirichenko et al., 2021). (c) Each gap junction is made up of two hemichannels, so called connexons. One hemichannel is formed by oligomerization of six connexin proteins. These can either be of the same type, forming a homomeric connexon, or of different types forming a heteromeric connexon. Further on, a gap junction is designated as homotypic if it is formed by two identical hemichannels and as heterotypic if two different hemichannels connect to each other (Meşe et al., 2007). (d) Two hemichannels forming a gap junction in the membranes of neighboring cells. The gap junction bridges a gap of 2–4 nm and allows for the specific flow passage of signaling molecules such as ATP, glutamate, and NAD^+^ (Evans et al., 2006; Thimm et al., 2005) (Figure created using biorender.com).

*GJB6* encodes the gap junction β‐6 protein or connexin 30 (Cx30), which is a subunit of a gap junction (Meşe et al., 2007). The GJB6 protein is expressed in the cell membrane and comprises four transmembrane domains with cytoplasmatic N‐ and C‐termini and a total length of 261 amino acids in humans and dogs alike (Kirichenko et al., 2021). The nucleotide substitution was predicted to lead to an exchange of an arginine to a tryptophan, XP_038429444.1:p.(Arg75Trp), in a position that is evolutionary conserved among mammals and birds. It affects the last amino acid of the first extracellular domain of the protein (Figure 4).

Multiple in silico tools predicted the *GJB6* variant to have a deleterious impact on the protein function. The SNPSs&GO software classified the effect as “Disease” (Calabrese et al., 2009). PredictSNP predicted a deleterious effect with an expected accuracy of 87% (Bendl et al., 2014) and MutPred2 gave a score of 0.718. Scores higher than 0.5 suggest pathogenicity (Pejaver et al., 2020).

## DISCUSSION

For this study we investigated a female Labrador retriever with an early onset paw pad hyperkeratosis affecting all four paws. At the time of sample taking for the genetic analysis, the dog was approximately 2 years old and otherwise healthy. WGS and filtering for private variants led to the discovery of a missense variant affecting the *GJB6* gene which is encoding a subunit of the gap junction.

Gap junctions are membrane spanning protein complexes that contain intercellular channels, allowing for communication between adjacent cells and maintaining cellular homeostasis (Wei et al., 2004). Each gap junction is made up of two interconnected hemichannels that are also referred to as connexons and are themselves formed by six connexins (Evans et al., 2006). These hemichannels are located in the membranes of two neighboring cells and enable the controlled exchange of ions, metabolites, and second messenger proteins (Scott et al., 2012).

Multiple different connexin proteins are known to be present in keratinocytes during the skin differentiation process. Among them is GJB6, which is expressed in skin and additionally also the brain and the inner ear (Di et al., 2005; Willecke et al., 2002). In humans, pathogenic *GJB6* variants have been associated with a form of hidrotic ectodermal dysplasia called Clouston syndrome. While this disease was first described in 1895 and more precisely defined in multiple members of a French‐Canadian family in 1929, the molecular basis was not discovered until much later in multiple affected members of a French family carrying a missense variant in the *GJB6* gene (Clouston, 1929; Lamartine et al., 2000). Clouston syndrome is an autosomal dominant disease and the main clinical signs in humans are sparse hair, nail dystrophy, and hyperkeratosis manifesting on the palms and soles at an early age (Cammarata‐Scalisi et al., 2019). Other *GJB6* variants have also been reported in patients with autosomal dominant and recessive deafness without any of the other symptoms that appear in the Clouston syndrome phenotype (del Castillo et al., 2002; Grifa et al., 1999). However, studies in knockout mice suggested that the deafness may not be directly caused by the deficiency of Gjb6, but rather the loss of connexin 26 encoded by *Gjb2*. *Gjb2* and *Gjb6* are neighboring genes with co‐regulated expression in mice and humans. A *Gjb6* ^*−/−*^ knockout strain that preserved Gjb2 expression had normal hearing (Boulay et al., 2013).

Differing from human patients with Clouston syndrome, the investigated Labrador Retriever had a normal fur coat and claws with only the paw pads showing variable degrees of hyperkeratosis. While the suspicion of an impaired hearing function was expressed by the owner, this was not further investigated. We therefore cannot make a clear statement as to whether the dog was affected by mild or more profound deafness. In the human ClinVar database, an entry with the homologous variant p.Arg75Trp was submitted for multiple human patients from one Chinese family and considered to be pathogenic (ClinVar accession: SCV002260213.4; Landrum et al., 2014). According to this entry, the family members are exclusively affected by autosomal dominant non‐syndromic hearing loss and therefore show a different phenotype than the dog we investigated for this study. Unfortunately, no detailed information about the patients is publicly available.

While the phenotypic differences of the Labrador Retriever reported herein to human patients with *GJB6* variants and Clouston syndrome are surprising, other genodermatoses are known, in which variants in the same gene cause somewhat different phenotypes in humans and dogs. One example is the *NSDHL*‐related CHILD‐syndrome in humans characterized by congenital hemidysplasia, ichthysosiform nevus and limb defects while dogs and cats with pathogenic *NSDHL* variants apparently only manifest the skin lesions, but not the severe limb malformations observed in human patients (Bauer et al., 2017; De Lucia et al., 2019; Happle et al., 1980; König et al., 2000).

To further categorize the identified variant in our case, we adapted the classification principles outlined in the Standards and Guidelines for the Interpretation of Sequence Variants by the American College of Medical Genetics and Genomics and the Association for Molecular Pathology (ACMG/AMP), acknowledging that these guidelines were originally designed for human diagnostics (Richards et al., 2015). The *GJB6* missense variant fit the following criteria for pathogenicity: PS1 (strong): same amino acid change as a previously established pathogenic variant—the dog described in this study caries the same variant as multiple human patients; PS2 (strong) de novo variant in a dog with both maternity and paternity confirmed and no family history; PM2 (moderate): absent from controls—the variant is not present in our 1664 control genomes or the other 90 genotyped Labrador retrievers; PP3 (supporting): multiple lines of computational evidence support a deleterious effect on the gene or gene product—as further described in the results section, the PredictSNP, SNPs&Go, and MutPred2 prediction tools all classified this variant as deleterious. With the accumulation of two strong, one moderate, and one supporting criteria, the variant XP_038429444.1:p.Arg75Trp was classified as pathogenic.

In conclusion, this study represents the first report of a pathogenic *GJB6* variant in an animal and therefore provides interesting new insights into the genotype–phenotype correlation of *GJB6* variants across different species. Further investigations are required to confirm these findings and to explain why the clinical phenotype of the studied Labrador Retriever differs from Clouston syndrome in humans.

## AUTHOR CONTRIBUTIONS

**Stefan J. Rietmann:** Investigation; writing – original draft; writing – review and editing; visualization. **Joseph Malatos:** Investigation; writing – original draft; visualization; writing – review and editing. **Vidhya Jagannathan:** Data curation; writing – review and editing. **Tosso Leeb:** Conceptualization; supervision; visualization; writing – original draft; writing – review and editing.

## FUNDING INFORMATION

This research was funded by the Swiss National Science Foundation, grant number 310030_200354.

## CONFLICT OF INTEREST STATEMENT

The authors declare no conflict of interest.

## ETHICS STATEMENT

The affected dog in this study was privately owned and blood and skin samples for diagnostic purposes were collected with the consent of the owner. The collection of blood samples from healthy control dogs was approved by the “Cantonal Committee for Animal Experiments” (Canton of Bern; permit BE94/2022).

## Supporting information


Table S1:

Table S2:

Table S3.

Table S4:

Table S5:

## Data Availability

The genome sequence data were submitted to the European Nucleotide Archive (ENA). All accession numbers are listed in Table S3.
